# Prevalence of putative virulence factors and antimicrobial susceptibility of *Enterococcus faecalis *isolates from patients with dental Diseases

**DOI:** 10.1186/1472-6831-8-17

**Published:** 2008-06-01

**Authors:** Randa Salah, Najla Dar-Odeh, Osama Abu Hammad, Asem A Shehabi

**Affiliations:** 1 Department of Pathology-Microbiology, Faculty of Medicine and Faculty of Dentistry, University of Jordan, Amman, Jordan; 2 Department of Oral Surgery-Medicine, and Periodontics, University of Jordan, Amman, Jordan

## Abstract

**Background:**

This study investigated the prevalence of *Enterococcus faecalis*, its putative virulence factors and antimicrobial susceptibility in individuals with and without dental diseases. A total of 159 oral rinse specimens were collected from patients (n = 109) suffering from dental diseases and healthy controls (n = 50).

**Results:**

*E. faecalis *was detected using only culture in 8/109 (7.3%) of the patients with various types of dental diseases, whereas no *E. faecalis *was found in the healthy controls weather using both culture and PCR. Phenotype characterizations of the 8 *E. faecalis *isolates indicated that 25% of the isolates produced haemolysin and 37.5% produced gelatinase. Most important virulence genes; collagen binding protein (*ace*) and endocarditis antigen (*efaA*) were present in all 8 *E. faecalis *isolates, while haemolysin activator gene (*cylA*) was detected only in 25% of isolates, and all isolates were negative for *esp *gene. All *E. faecalis *isolates were 100% susceptible to ampicillin, chloramphenicol, ciprofloxacin, vancomycin, and teicoplanin, and to less extent to erythromycin (62.5%).

**Conclusion:**

This study shows that all *E. faecalis *isolates were recovered only from patients with dental diseases especially necrotic pulps, and all isolates carried both collagen binding protein and endocarditis antigen genes and highly susceptible to frequently used antimicrobial drugs in Jordan.

## Background

Many studies demonstrated that *E. faecalis *is frequently found in patients suffering from oral infections like gingivitis, periodontitis, teeth with failed endodontic as well as acidic carious lesions associated with persistently infected root canals. [1-4] Other studies demonstrated the frequent presence of *E. faecalis *in association with a wide variety of aerobic and anaerobic bacterial species involved in various endodontic diseases and chronic apical periodontitis [4-7].

Virulent factors of *E. faecalis *include adherence to host tissue, invasion and abscess formation, modulation of host inflammatory responses, secretion of various products which enhances biofilm formation [8-10]. Data on oral prevalence of *E. faecalis *and its virulence factors vary from one study to another [1,6,7]. Therefore, more investigation on potential virulence factors of *E. faecalis *would be useful in understanding their role in dental infections. Moreover, clinical isolates of *E. faecalis *recovered from root canal infections can express antimicrobial resistance to conventional treatment regimens recommended for dental procedures [11-13].

This study aimed to investigate the occurrence of *E. faecalis*, its virulence factors and antimicrobial susceptibility in association with and without some dental diseases in a Jordanian population.

## Results

**Figure 1:**
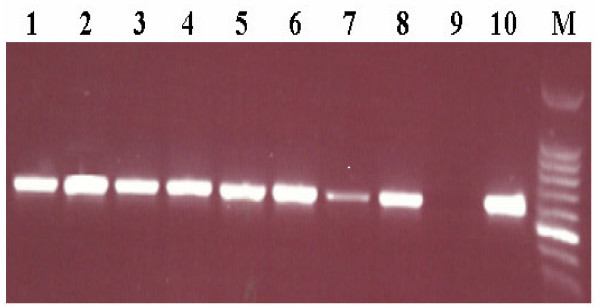
**Distribution of *efaA *gene among the 8 oral *E. faecalis *isolates.** M, 100 bp DNA marker; Lane 1, *efaA *(688 bp); positive control (*E. faecalis *ATCC 29212); Lane 2, *efaA *negative control; Lanes 3 to 10, positive to *efaA *among oral *E. faecalis *isolates.

Age of the patients ranged from 14 to 75 years (mean; 36.4 year), and the control persons were between 20 and 69 years (mean; 28.3 year). The prevalence of *E. faecalis *isolates in oral rinse specimens of patients with dental diseases was 8/109 (7.3%) using both culture and PCR tests, whereas all oral rinse specimens obtained from the 50 healthy control persons were negative for *E. faecalis *using both methods. All DNA extracted from 8 *E. faecalis *isolates were proved to be positive for specific *E. faecalis *16s rRNA gene. The difference between the results of the two groups is statistically significant (P value = 0.031). In addition, there was 2 *E. avium *isolates (Table 1). The growth pattern of *E. faecalis *isolated from positive cases varied from few to numerous colonies (2 – 50 × 10^3 ^colonies/ml). The distribution of 8 *E. faecalis *isolates among patients in association of sex, smoking, oral hygiene and dental diseases is shown in (Table 2). Detection of putative virulence factor genes among 8 *E. faecalis *isolates using PCR were as follows; both *ace *and *efaA *genes were present in all isolates (100%), while *cylA *gene was detected only in 2 isolates, and all isolates were negative for *esp *gene (Table 3, Figure 1). All 8 *E. faecalis *isolates were 100% susceptible to, ampicillin, chloramphenicol, ciprofloxacin, teicoplanin and vancomycin, while 62.5% and 12.5% of the isolates were susceptible to erythromycin, imipenem, respectively, and all were resistant to gentamicin, clindamycin and Oxacillin

**Table 1:** Detection of *Enterococcus species *in patients and controls

Characteristics	109 Patients with dental diseases No. (%)	50 Controls persons No. (%)
Age mean (years)	36.4 ± 13.98	28.3 ± 12.59
Sex		
Male	39 (36)	24 (48)
Female	70 (64)	26 (52)
*E. faecalis*	8 (7.3)	0
*E. avium*	2 (1.8)	0

**Table 2:** Distribution of 8 *E. faecalis *isolates in association of sex, smoking, oral hygiene and dental diseases among 109 patients

Characteristics	Positive E. faecalis*	Negative E. faecalis*	Total No.	P-value***
Female	7	63	70	0.134
Male	1	38	39	
Nonsmoker	7	83	90	0.228
Smoker	1	18	19	
Poor Oral hygiene	7	68	75	0.222
Good Oral hygiene	1	33	34	
Gingivitis +ve	7	64	71	0.161
Gingivitis -ve	1	37	38	
necrotic pulps	8**	101	109	0
caries +ve	4**	77	81	0.115
Caries -ve	4	24	28	

*colony count: 2 – 50 × 10^3^colonies/ml.

** Each one patient was treated with antibiotics during the last month.

*** Not significant between positive *E. faecalis *and each of sex, smoking, oral hygiene, gingivitis, caries but significant with necrotic puples.

**Table 3:** Presence of 16S rRNA gene and prevalence of *esp, cylA, ace, efaA *genes among the 8 oral *E. faecalis *isolates detected by PCR.

Isolate No.	16S rRNA	*esp*	cylA*	*ace*	*efaA*
3	+	-	-	+	+
44	+	-	-	+	+
1	+	-	-	+	+
2	+	-	+	+	+
6	+	-	-	+	+
12	+	-	-	+	+
18	+	-	-	+	+
59	+	-	+	+	+
Total strains No. (%)	8 (100%)	0 (0.0%)	2 (25%)	8 (100%)	8 (100%)

*Two isolates expressed hemolytic activity in vitro

## Methods

A total of 159 subjects who were attending the Dental clinic/Jordan University Hospital (JUH), Amman, Jordan were examined for presence of dental diseases by two dentists (N. Dar-Odeh & O. Abu Hammad) during the study period of 2005. Subjects were divided into two groups according to the presence or absence of dental diseases. Control group consisted of 50 healthy persons who do not have any obvious dental disease. Dental diseases investigated include: dental caries, plaque-induced gingivitis, and root canal disease. Personal data, smoking history, presence or absence of clinical dental disease, oral hygiene and antibiotic treatment during the last month were recorded for each examined person. Dental caries and gingivitis were recorded as present/absent. Root canal disease included: irreversible pulpitis, necrotic pulp only, and periapical periodontitis. Oral hygiene was considered as either poor or good according to plaque index. All patients and control persons had given their written consent to be included in the study. All patients and controls were asked to rinse their mouths for 60 seconds with 10 ml sterile distilled water and returned the oral rinse to a sterile container.

### Specimens Processing

All specimens were transferred immediately to the Microbiology Research Laboratory/Faculty of Medicine/University of Jordan. Oral rinse specimens were poured into sterile tube and centrifuged for 10 min at 10,000 g. The supernatant discarded and the remaining pellets were re-suspended by vortex in one ml of sterile normal saline 0.9% [14].

### Isolation of Enterococcus species

A loop full of the suspended pellets (0.01 ml) was cultured on bile-esculin agar plates to detect and count the presence of gray to black colonies of *Enterococcus species*. The rest of the suspended specimens were stored at -70°C for further investigations. At least three colonies grown on bile-esculin plates were sub-cultured into blood agar plates to isolate *Enterococcus species *(Oxoid, England). All cultures carried out through the study were incubated in a candle jar (5% CO_2_) for 24–48 hrs at 37°C. Pure growth obtained from blood agar plates was again inoculated onto Cysteine Lactose Electrolyte Deficient (CLED) agar plates (Oxoid, England), 6.5% NaCl solution and bile-esculin tube agar. Every growth showing gram-positive cocci, positive bile-esculin, positive 6.5% NaCl tests, catalase-negative and appearing as yellow, small/medium in size on CLED agar (Oxoid, England) were recorded tentatively as *Enterococcus *isolates. *E. faecalis *ATCC 29212 was included as a positive control through out the study.

### Biochemical detection of E. faecalis isolates

All tentative *Enterococcus *isolates were subcultured on brain heart infusion agar plates (Oxoid, England) and tested using biochemical Remel system (RapID™ STR system, USA) to confirm their identity as *E. faecalis*.

### Hemolysin and gelatinase activities

*E. faecalis *isolates were assessed for hemolytic activity on blood base agar (Oxoid, England) supplemented with 5% (v/v) human blood. A single colony was cultured onto blood agar plates and its hemolytic activity was determined by presence of clear zone around the colonies (β hemolysis) as reported by Creti *et al*. [15]. Gelatinase activity was assessed by inoculation of single colonies of each isolate as spot form on 12% gelatin plates (Defico, USA). The plates were incubated at room temperature for 48 hrs. Gelatinase activity was evident by the presence of liquefied zone around the colonies. *Serratia mercenses *ATCC 13880 was used as a positive control for gelatinase production test.

### Antimicrobial susceptibility test

Susceptibility of *E. faecalis *isolates to 9 antimicrobial agents was determined using disc diffusion method according to NCCLS (now CLSI) guidelines [16].

### Detection of E. faecalis in oral rinse sample by PCR

DNA extraction was performed using Wizard Genomic DNA Purification Kit (Promega, USA), according to manufacturer's instructions. The DNA extractions were used for detecting the specific gene 16S rRNA of *E. faecalis *in oral rinse samples [17]. PCR conditions were accomplished by a PCR thermocycler (MJ research- INC, USA), and were as follows: 15 min initial enzyme activation/DNA denaturation step at 95°C followed by 35 consecutive cycles at 94°C for 20 s; 68°C for 45 s; 72°C for 15s. PCR products were analyzed by electrophoresis using 2% agarose gel (Promega, USA) containing ethidium bromide in 1× TBE buffer, and run for 1 hr with 70V, and visualized by UV Trans-illuminator (UVP) and Gel Documentation System (UVP). The same PCR reaction was tested with each single control strain of *E. faecium *and *S. mutans *which were isolated from other clinical specimens at JUH, and by using *E. faecalis *ATCC 29212.

The PCR test used, gave a negative result with DNA extracted from *E. faecium *and *S. mutans*. Also, DNA extraction of recovered and identified *E. faecalis *isolates were confirmed using the same DNA extraction and PCR procedure under the same conditions used for oral rinse specimens (Table 4).

**Table 4:** Antimicrobial susceptibility of 8 *E. faecalis *by disc diffusion method

Antimicrobial agents	No. (%) of isolates susceptible
Ampicillin	8 (100)
Chloramphenicol	8 (100)
Ciprofloxacin	8 (100)
Teicoplanin	8 (100)
Vancomycin	8 (100)
Erythromycin	5 (62.5)*
Gentamycin	Null
Clindamycin	Null
Oxacillin	Null

Harbored only *ace*, *efaA *genes.

### Detection of E. faecalis putative virulence genes

DNA of *E. faecalis *isolates was prepared by suspending a loop full of overnight growth colonies grown on blood agar in a tube that contained 500 μl sterile distilled water, followed by boiling for 10 min and then centrifuged at 12,000 g for 6 min. An aliquot of the supernatant (5 μl) was used as the template in a final volume of 25 μl PCR mixture [15]. PCR amplification for the following genes: collagen binding protein (*ace*), endocarditis antigen (*efaA*), haemolysin activator (*cylA*), and a surface protein (*esp*) were prepared as uniplex in a 25 μl final reaction volume. Table 5 shows the primers used in the study [15,17,18].

**Table 5:** *E. faecalis *primers used in the study

**Gene**	**Sequence**	**Product size (bp)**	**Reference**
*E. faecalis *16S rRNA	Ef16SF 5' – CCGAGTGCTTGCACTCAATTGG – 3'	138	17
	Ef16SR 5' – CTCTTATGCCATGCGGCATAAAC – 3'		
Esp	5'-TTGCTAATGCTAGTCCACGACC-3'	932	18
	5'-GCGTCAACACTTGCATTGCCGA-3'		
cylA	5'-GACTCGGGGATTGATAGGC-3'	688	15
	5'-GCTGCTAAAGCTGCGCTTAC-3'		
ace	5'-GGAATGACCGAGAACGATGGC-3'	616	15
	5'-GCTTGATGTTGGCCTGCTTCCG-3'		
efaA	5'-GCCAATTGGGACAGACCCTC-3'	688	15
	5'-CGCCTTCTGTTCCTTCTTTGGC-3'		

Samples were amplified in a PCR thermal cycler (MJ research- INC, USA), by heating for 5 min at 95°C, followed by 30 cycles of 95°C for 60 s, 58°C for 60 s (63°C for *esp*) and 72°C for 60 s, and a final step of 72°C for 10 min. PCR products were analyzed in 0.8% agarose gel electrophoresis (containing 0.5% ethidium bromide in 1× TBE buffer) which run for 50 minutes by 80 voltages using horizontal electrophoresis apparatus, and visualized by Gel Documentation System (UVP, USA). *E. faecalis *ATCC 29212 was used as a positive control in each PCR run to detect *cylA*, *ace*, *efaA *genes, and to test DNA prepared from certain *E. faecalis *strains (EFS121, EFS87, EFS16, EFS27B, EFSU85, and EFS118) which were obtained from Dr. Roberta Creti (Dipartimeno Di Malattie Infecttive, Parassitarie ED Immunomediate, Istituto Superiore di Sanità, Viale Regina Elena, 299–00161 Rome, Italy). These DNA preparations were used as a positive control for *esp *gene along with 1 Kb/100 bp Ladder marker (promega, USA).

### Statistical analysis

Z test was used to compare the prevalence of *E. faecalis *in patients group and control group according to the following equation:

Z = P_1 _- P_2_/√P(1-P) (1/n_1 _+ 1/n_2_). *P < 0.05 *was considered statistically significant.

## Discussion

The present study shows that *E. faecalis *isolates were recovered from Jordanian patients in association with one or more of the following dental diseases; caries, gingivitis, plaque-induced gingivitis, and endodontic infection, and in a significant rate (7.3%; P = 0.031) compared to healthy control persons (Zero). Despite the fact that generally PCR is more sensitive than culture method in detection of bacteria in clinical specimens, this study has not detected any positive *E. faecalis *using direct oral rinse specimens. Sedgley CM et al., 2005 (19) found that a quantitative real-time PCR reported a higher incidence of *E. faecalis *in oral rinse samples than culture techniques and afforded greater sensitivity. The study revealed that poor oral hygiene, gingivitis and necrotic pulps appear to be important predisposing factors for infection with *E. faecalis *(Table 2). A recent similar study from USA has isolated *E. faecalis *from 11% of oral rinse samples of patients receiving endodontic treatment, but only 1% of *E. faecalis *was recovered from dental students with no history of endodontic treatment [14]. Enterococci are able to colonize the oral cavity, particularly in patients with periodontitis or root canal infections associated with oral mucosal lesions and in immuno-compromised patients [20,21]. Also, *E. faecalis *is the most commonly isolated species from root canals samples with endodontic failure [2,21,22]. *E. faecalis *has been often isolated in pure culture or as a predominant organism in previously root- filled teeth with periapical lesions or chronic apical periodontitis [3,19,23,24]. Furthermore, it has been found that this organism persistently infected root canal where calcium hydroxide medication is ineffective [25].

This study shows that the production of hemolysin and gelatinase as putative virulence determinants were not always expressed by *E. faecalis *isolates in association with dental diseases, since only 25% of *E. faecalis *isolates expressed hemolysin and 37.5% gelatinase activity in vitro, respectively. Two studies carried by Sedgley *et al*. [14,23] reported different results with hemolysin production, first study has proved that 36% of the *E. faecalis *strains recovered from endodontic patients produced hemolysin, while the second has not detected production of hemolysin in any enterococcal isolates from endodontic cases. Also, Sedgley *et al*. [22] found that gelatinase gene (gelE) was detected in all endodontic isolates of *E. faecalis *while expressed gelatinase activity was observed in two thirds of the isolates. These studies concluded that evidence of potential virulence factors were identified in endodontic *Enterococcus spp*., specifically production of gelatinase and response to pheromones. Other studies indicated that expression of gelatinase gene contributed to the increased dissemination of *E. faecalis *in high-density environments and was associated with increased adhesion of *E. faecalis *to dentine *in vitro *[26,27].

The present study has shown that both *ace *and *efaA *genes were present in all *E. faecalis *isolates, while *cylA *gene was detected only in two isolates, and all isolates were negative for *esp *gene which is mostly found in *E. faecalis *strains isolated from urinary tract infections [18].

A recent molecular-based study indicated that virulence determinants *efaA *and *ace *genes has been found in *all E. faecalis *isolates from root canal of endodontic patients, whereas *esp *gene was present in (58%) and *cylA *gene in (19.4%) of the isolates [11]. These results are in agreement with our results except for *esp *gene. In general, the expression of hemolysin and gelatinase activity or their genes along with the occurrence of other virulence genes (*esp*, *cylA*, *ace*, *efa A*) in *E. faecalis *strains varied widely from one study to another and probably due to the difference in their clinical and geographic origins [5,9,11,26-29]. Furthermore, neither esp nor gelatinase seemed to be required for biofilm formation; both *E. faecalis *and *E. faecium *did not show a correlation between the presence of either esp or the production of gelatinase and biofilm formation [30]. It appears that many environmental and genetic factors may be associated with the production of biofilm by *E. faecalis *[31].

In recent years, enterococci have received increasing attention because of the development of resistance to multiple antimicrobial drugs and its common prevalence in nosocomial infections. Vancomycin-resistant enterococci (VRE) probably represent currently the most serious challenge among many microbes with antibiotic resistance causing human infections [32]. Our results showed that all *E. faecalis *isolates were susceptible to chloramphenicol, ampicillin, vancomycin, ciprofloxacin, and teicoplanin, but the isolates were much less susceptible to erythromycin. A study by Pinheiro *et. al*. [12] showed that *E. faecalis *isolates from oral specimens were completely susceptible *in vitro *to amoxicillin, amoxicillin-clavulanic acid, vancomycin and to less extent susceptible to erythromycin, moxifloxacin, chloramphenicol, tetracycline, doxycycline, and ciprofloxacin. The susceptibility result of our limited number of *E. faecalis *isolates indicated higher frequency of resistance rates than those reported in recent studies from western countries [11-13]. Moreover, our susceptibility result correlate well with the high prevalence of resistance among clinical and community *Staphylococcus aureus *isolates in Jordan [33].

In conclusion, this study shows that all *E. faecalis *isolates are associated with various dental diseases especially necrotic pulps in patients and they carried both collagen binding protein and endocarditis antigen genes.

## Competing interests

The authors declare that they have no competing interests.

## Authors' contributions

AAS and ND-O contributed to the design of all experiments and writing the manuscript. ND-O and OAH examined all patients and control persons and collected the oral specimens. RS performed all laboratory tests and co-wrote the manuscript.

## Pre-publication history

The pre-publication history for this paper can be accessed here:


